# Arrhythmogenic Right Ventricular Cardiomyopathy: Improvement of Diagnosis and Risk Stratification by Implementation of Echocardiographic Normative Values in Children

**DOI:** 10.3389/fped.2022.868791

**Published:** 2022-03-22

**Authors:** Stefan Kurath-Koller, Martin Koestenberger

**Affiliations:** Division of Pediatric Cardiology, Department of Pediatrics, Medical University Graz, Graz, Austria

**Keywords:** arrhythmia, arrhythmogenic right ventricular cardiomyopathy (ARVC), echocardiography, risk assessment scoring system, right ventricular function

## Introduction

A recent review on arrhythmogenic right ventricular cardiomyopathy (ARVC) in pediatric patients highlights the diagnostic challenge and its important therapeutic consequences in this rare disease (1). ARVC leads to fibro-fatty infiltration of predominantly the right ventricular (RV) myocardium and typically manifests beyond 10 years of age, causing arrhythmias and sudden cardiac death. For diagnosis, the “Task Force Criteria” (2) are used, defining major and minor criteria from 2D echocardiography, cardiac magnetic resonance imaging (MRI), tissue characterization, electrocardiogram (ECG), RV angiography, arrhythmias, and family history. An international expert report published in 2020 (3) incorporates clinical variants of ARVC, i.e., “biventricular disease variants” and “the LV phenotype characterized by isolated LV involvement,” and constitutes a critical appraisal of the 2010 “Task Force Criteria.” Echo and MRI criteria include right ventricular outflow tract (RVOT) dimension and RV function parameters. However, these “Task Force Criteria” were designed for patients above 14 years of age and do not incorporate pediatric normative values. RVOT dimensions, for example, are assessed as mm/m^2^ body surface area (BSA) derived from the parasternal short (PSAX) and parasternal long axes (PLAX). For pediatric patients, the cut-off values for RVOT dimensions included in the “Task Force Criteria” are not appropriate. However, normative values for the RV size and for RVOT dimensions in children exist (4, 5).

## Discussion

We think that normative values for RV size and RVOT dimension (4, 5) should be incorporated for diagnosis of ARVC in patients <14 years of age. A diagnosis of ARVC may not be made very often in this age group but is particularly difficult to establish in suspicious patients. Echocardiographic major and minor criteria might be adapted to pediatric normative values. Certainly, this warrants further research to prove the efficiency of such adaptations.

Also, RV function in pediatric patients is generally well studied, and normative function parameter values exist for relevant echocardiographic variables, such as tricuspid annular plane systolic excursion (TAPSE), tricuspid annular peak systolic velocity (S′), or the subcostal derived TAPSE (S-TAPSE) (6, 7). Future pediatric studies in our opinion should incorporate these normative values for possible use in ARVC diagnosis. The available normative values of the RVOT size in the pediatric age group (4), when compared to expected enlarged RVOT values in children with, e.g., arrhythmogenic right ventricular dysplasia (ARVD), will significantly improve the diagnostic criteria for children with suspected ARVD.

Recently, novel diagnostic criteria for arrhythmogenic cardiomyopathy have been proposed, with the incorporation of late gadolinium enhancement (LGE) as a diagnostic criterion (8). LGE is potentially useful for diagnostic purposes and prediction of future disease progression in ARVC gene carriers (9). ARVC patients can be risk stratified into high, intermediate, or low risk, carrying at least 10%, 1–10%, or <1% risk for sudden cardiac death (SCD) per year. High-risk criteria comprise aborted SCD due to ventricular fibrillation (VF), sustained ventricular tachycardia (VT), and severe RV/left ventricular (LV) dysfunction. In high-risk patients implantable cardiac defibrillator (ICD) implantation is recommended (class I indication). RV/LV function also plays a role for intermediate risk classification. In patients with moderate RV/LV dysfunction, ICD implantation is a class Ia indication (10). Moderate RV dysfunction is determined by RV fractional area change between 24 and 17% or RV ejection fraction (EF) between 40 and 36%. However, in pediatric patients, RV fractional area change (FAC) is difficult to assess with echocardiography. Therefore, other values, such as TAPSE, S-TAPSE, or S′ in our opinion should be incorporated into the assessment of intermediate risk and ICD implantation indication in patients <14 years of age to improve the accuracy of RV function determination by echocardiography. ICD implantation in children may not be performed frivolously, especially given the fact that serious short- and long-term complications may arise.

The currently used “Task Force criteria” were last updated in 2010 (2); therefore, future editions might include new parameters for diagnosis and eventually risk stratification in patients below 14 years of age. Herein we would like to raise awareness inside the community for the potential value of implementation of pediatric normative values for risk stratification and diagnosis of ARVC in children.

## Author Contributions

SK-K and MK drafted and edited the manuscript. Both authors approved the manuscript in its current form prior to submission.

## Conflict of Interest

The authors declare that the research was conducted in the absence of any commercial or financial relationships that could be construed as a potential conflict of interest.

## Publisher's Note

All claims expressed in this article are solely those of the authors and do not necessarily represent those of their affiliated organizations, or those of the publisher, the editors and the reviewers. Any product that may be evaluated in this article, or claim that may be made by its manufacturer, is not guaranteed or endorsed by the publisher.
